# Lenacapavir-Based Salvage Therapy in Heavily Treatment-Experienced Multidrug-Resistant HIV-1: A Case Report From the United Arab Emirates

**DOI:** 10.7759/cureus.115988

**Published:** 2026-09-09

**Authors:** Zartash Z Khan, Nidal Hassan

**Affiliations:** 1 Infectious Diseases, Dubai Health, Dubai, ARE; 2 Infectious Diseases, Zayed Military Hospital, Abu Dhabi, ARE

**Keywords:** heavily treatment-experienced, hiv-1, lenacapavir, multidrug-resistant hiv, uae

## Abstract

Management of heavily treatment‑experienced people with HIV remains challenging due to cumulative drug resistance and limited therapeutic options. We report the case of a cisgender female with HIV‑1 subtype A1 and extensive antiretroviral therapy (ART) resistance who experienced more than a decade of suboptimal viral suppression despite multiple ART regimens. The patient accumulated resistance to all nucleoside/nucleotide reverse transcriptase inhibitors (NRTIs), all non‑nucleoside reverse transcriptase inhibitors (NNRTIs), and nearly all protease inhibitors (PIs), with limited remaining treatment options. Following virologic rebound in 2023 and failure of subsequent salvage regimens, lenacapavir was initiated in 2024 in combination with an optimized background regimen of bictegravir/emtricitabine/tenofovir alafenamide (BIC/FTC/TAF). The patient rapidly achieved an undetectable viral load and remains virologically suppressed to date.

## Introduction

Advances in antiretroviral (ARV) drug development have significantly improved outcomes in mortality and morbidity associated with HIV infection; however, treatment of HIV with multiple mutations requires a personalized approach [1, 2]. Consequently, considering treatment and management of heavily treatment-experienced (HTE) patients, providers must have a detailed understanding of prior resistance mutations, the significance of such mutations, and the underlying processes that caused previous treatment failures, as well as corresponding ARV pharmacology profiles to leverage the choice of appropriate ARV [3].

On the other hand, the introduction of ARVs with novel mechanisms of action and higher resistance barriers is also essential for expanding access to effective HIV therapy [4]. The first-in-class selective inhibitor of HIV capsid protein lenacapavir is an example of such agents. Lenacapavir retains antiviral activity against HIV isolates harboring resistance-associated mutations against nucleoside/nucleotide reverse transcriptase inhibitors (NRTIs), nonnucleoside reverse transcriptase inhibitors (NNRTIs), protease inhibitors (PIs), and integrase strand transfer inhibitors (INSTIs), i.e., in the case of resistance against any of the major ARV classes [5].

Reports on real-world management of multidrug-resistant HIV are still scarce. While lenacapavir has proven to be effective for this genotype in clinical trial settings [5], such success should be mirrored by real-world evidence. This report describes a treatment journey of over a decade for a patient with multidrug-resistant HIV-1, with all available genotype resistance test (GRT) panels documented and outcomes of treatment strategies utilized prior to initiation of lenacapavir, along with an optimized background regimen.

## Case presentation

A 68-year-old cisgender female with an initial presentation of cryptosporidium gastroenteritis and esophageal candidiasis who was diagnosed with AIDS, with HIV-1 subtype A1 infection, with multiple mutations present on genotypic resistance testing. The patient was subsequently diagnosed with cervical cancer, vulvar Bowen's disease, thyroid cancer, HPV associated genital warts, recurrent herpes simplex virus type 2 genital infection, and severe osteoarthritis. The actual mode of acquisition of HIV was not determined. The patient had reported emergency blood transfusion following a motor vehicle accident that led to an emergency abortion in India in 1998; however, behavioral risk history and spouse's HIV status were unclear.

The patient was initially diagnosed with HIV reportedly in 2004. Details on initial viral load, genotype, immunological status, and antiretroviral therapy were not accessible. She was non-adherent with ARV and had lost follow-up with medical care up until 2013, at which time she was hospitalized with profound diarrheal enteropathy and found to have AIDS, with laboratory tests showing an absolute CD4 count of 18 cells/µL and a CD4 percentage of 2%, and a viral load (VL) of 288,099 copies/mL. The baseline available genotypic resistance test (GRT) demonstrated multidrug resistance with major and accessory mutations against NRTIs (M184V; T215F/I/S; K219Q), NNRTI (K103N; V179I), and PIs (I47A/V; V82I; L89V); additionally, there were accessory mutations against INSTIs (L74I; G134N; S283G), along with various polymorphic mutations. All accessible GRT results are available in Table 1. ARV treatment was delayed based on the treatment guidelines, as the patient had presented with AIDS and was found to have opportunistic infections with cryptosporidiosis and esophageal candidiasis, while active tuberculosis was investigated and subsequently ruled out. Additionally, establishment of care was to be confirmed prior to ARV treatment initiation. 

**Table 1:** Serial GRT results Bold formatting indicates the mutations associated with reduced susceptibility or virological response either alone or in combination with other mutations. GRT - genotypic resistance test; NRTI - nucleoside/nucleotide reverse transcriptase inhibitor; NNRTI - non-nucleoside reverse transcriptase inhibitor; PI - protease inhibitor; INSTI - integrase strand transfer inhibitor

Date	NRTI	NNRTI	PI	INSTI
Year 2013 (considered as baseline)	M184V, T215F/I/S, K219Q, V118I	K103N, V179I, E219D, Q102K	I47A/V, V82I, I13V, L19T, K20R, V32I, L33I, E35D, M36I, R41K, R57K, L63P, H69K, L89V	K14R, S17N, V31I, V32I, S39N, L63I, V72A/I/T, L74I, T112I, T124A, T125A, V126F, G134N, I135V, K136, D167E, A169V, K173R, V201I, I203M, N222K, V259I, S283G
	E6D, V35T, K49R, V60I, K122E, D123S, I135T, C162S, S168C, T165L, K173Q, Q174K, D177E, T200A, Q207A, R211S, F214L, V245E, D250E, R277K, T286A, P294T, E297Q, V317A, G335D, I341V, R356K, M357K, K358R, G359S, T369A, A371V, I375V, A376V, T377L, T386S, K388R, E396D, A400T			
2 years from baseline	M184V, T215F, K219Q	E138A/G, Y181I	None reported	None reported
3 years from baseline	D67N, K70R, M184V, T215F/I/L/S, K219Q	E138A/G, Y181I	V32I, I47A/V, A71V, L89V	None reported
4 years from baseline	D67N, K70R, M184V, T215F/I/S, K219Q	K103N, E138A, Y181I	V32I, I47A/V, A71V, L89V	None reported
10 years from baseline	None reported	K103N	V32I, I47V, G73S, L89M/V	L74M
10 years and 4 months from baseline	M184V, D67N, K70R, T215F/I/L/S, K219Q	K103N, Y181I, E138A	V32I, I47A/V, A71V, G73S, L89M/V	L74M

The patient was scheduled to receive the first combination antiretroviral therapy (ART) of tenofovir disoproxil fumarate/emtricitabine (TDF/FTC) + etravirine (ETR) + raltegravir (RAL) about a month after diagnosis; however, due to issues with logistics in association with patient follow-up and timely availability of ETR, treatment initiation was delayed by nearly 11 months. The ART was interrupted one month after initiation due to a severe HIV enteropathy-induced diarrhea. Other opportunistic infections contributing to enteropathy were investigated and excluded. Immunologic and virologic monitoring tests four months later showed a VL of 18,730 copies/mL and a CD4 count of 139 cells/µL. A year from ART initiation, follow-up GRT revealed mutations against NRTIs and NNRTIs (Table 1), with no significant mutations against PIs or INSTIs. At this point, the patient's ART was switched to TDF/FTC in combination with lopinavir/ritonavir (LPV/r), and ETR was stopped due to the Y181 mutation; RAL was not continued for an unclear reason. On follow-up one year after the last adjustment in ART, HIV viremia persisted, though RNA levels decreased to 3930 copies/mL, with a CD4 count of 265 cells/µL. About then, the patient traveled to Germany to receive chemotherapy for cervical cancer, where another GRT was performed (results unavailable), which prompted a change in the ART to tipranavir/ritonavir (TPV/r) + maraviroc (MVC) + enfuvirtide. Over the subsequent year, the patient's treatment was interrupted intermittently due to enfuvirtide intolerance as well as delayed and interrupted access to maraviroc.

Later that year, the GRT revealed the emergence of new mutations against PIs in addition to mutations against NRTI and NNRTIs, and dual tropism. The ART regimen was then adjusted to double-dose TDF + efavirenz (EFV)+ TPV/r + maraviroc. The choice of EFV in the presence of archived K103N and MVC, in view of dual tropism in the ART, was then unclear. A year after the last ART adjustment, a VL of 781 copies/mL was detected, and the GRT demonstrated resistance to all NRTIs, all NNRTIs, and all PIs except for the TPV/r and atazanavir/ritonavir (ATZ/r) combinations; hence, no switch in ART was employed. The decline in VL continued for the next few years, until it reached undetectable levels in 2021, with a CD4 count of more than 500 cells/µL. The viral suppression was then maintained for another two years, with the latest undetectable VL recorded at the beginning of 2023, coupled with a CD4 count of more than 900 cells/µL.

However, a few months later, despite reported adherence to treatment, the virological analysis returned a VL of 114 copies/mL, and she also developed recurrent anal warts. The reported genotypic analysis contained TPV mutations and dual tropism. Accordingly, ART was switched to doravirine/lamivudine/TDF (DOR/3TC/TDF) + darunavir/ritonavir (DRV/r) + fostemsavir. However, monitoring tests about six months after the change in ART detected a VL of 19,800 copies/mL and a CD4 count of 454 cells/µL, in addition to mutations against NNRTIs, PIs, and INSTIs, and an invalid tropism assay result. No change in ART was recorded. The patient's recorded VL measurements and CD4 counts are demonstrated in Figure 1 and Table 2, respectively.

**Figure 1:**
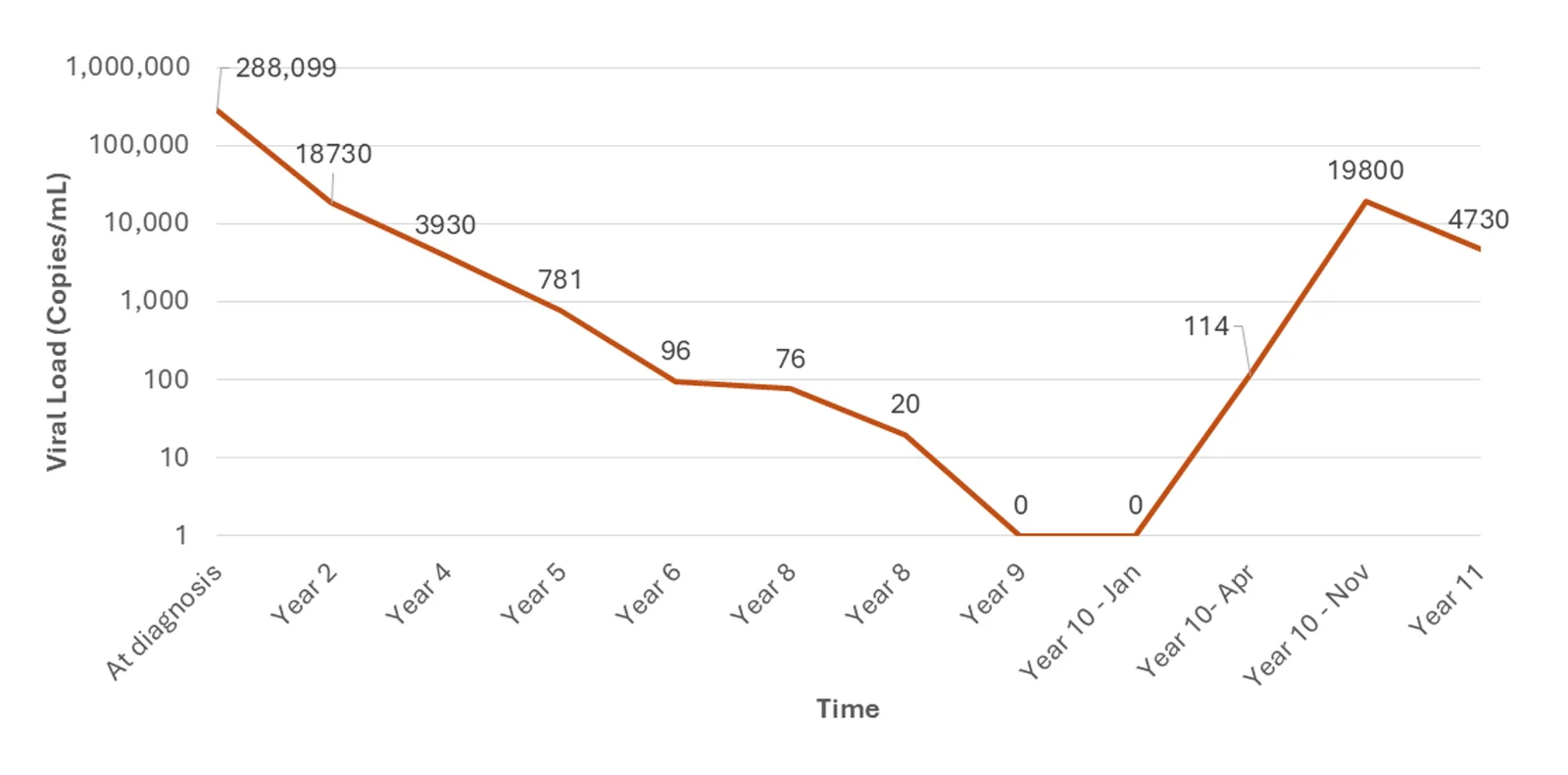
Change in the patient's viral load in the course of ten years prior to lenacapavir initiation

**Table 2:** Recorded CD4 counts for the patient in the course of ten years prior to lenacapavir initiation *Recorded at the time the patient presented to our center

Time	CD4 count	Normal range
Year 1	18 cells/µL	500-1200 cells/µL
Year 2	139 cells/µL	
Year 4	265 cells/µL	
Year 9	>500 cells/µL	
Year 11	>900 cells/µL	
Year 11	454 cells/µL	
Year 12*	368 cells/µL	

The patient presented in January 2024 with a VL of 4730 copies/mL and a CD4 count of 368 cells/µL, while on DOR/3TC/TDF +DRV/r. The patient was not able to continue fostemsavir due to reported pruritus. GRT showed resistance to numerous NRTIs, NNRTIs, and PIs, as well as dual tropism. We reviewed the potential ART options with mechanisms of action not compromised by the patient's resistance profile. Fostemsavir was not considered an option in view of previously reported intolerance; additionally, it was not available in the UAE at that time, and ibalizumab administration was not feasible for the patient. Other investigational agents, including leronlimab (PRO-140), semzuvolimab (UB-421), and islatravir, were neither available nor approved at that time. Accordingly, lenacapavir, along with an optimized background of bictegravir/emtricitabine/tenofovir alafenamide (BIC/FTC/TAF), was selected as a salvage therapy for this patient.

The patient was initiated on BIC/FTC/TAF, with a request submitted for lenacapavir. In one month, the patient's viral load had declined to less than 40 copies/mL. In the month following, the viral load was 52 copies/mL, and lenacapavir, which by then was available, was initiated (two 463.5-mg subcutaneous injections, administered periumbilically after oral lenacapavir 600 mg (300-mg tablets ×2) on days one and two). Subsequent monitoring showed a viral load of 36 copies/mL and a CD4 count of 469 cells/µL. Five months from ART with BIC/FTC/TAF and lenacapivir a sustained virological response with a viral load of less than 40 copies/mL, with a CD4 count of 558 cells/µL, was achieved. By the following month, the viral load became undetectable, and the patient received the second lenacapavir dose (two 463.5-mg subcutaneous injections, administered periumbilically). The patient has since continued BIC/FTC/TAF daily with lenacapavir subcutaneously every six months and remains virologically suppressed with sustained immunologic recovery. The regimen was well-tolerated with only a persistent but non-tender injection-site nodule observed; no cardiac abnormalities or compliance issues were reported. The patient's follow-up journey is illustrated in Figure 2.

**Figure 2:**
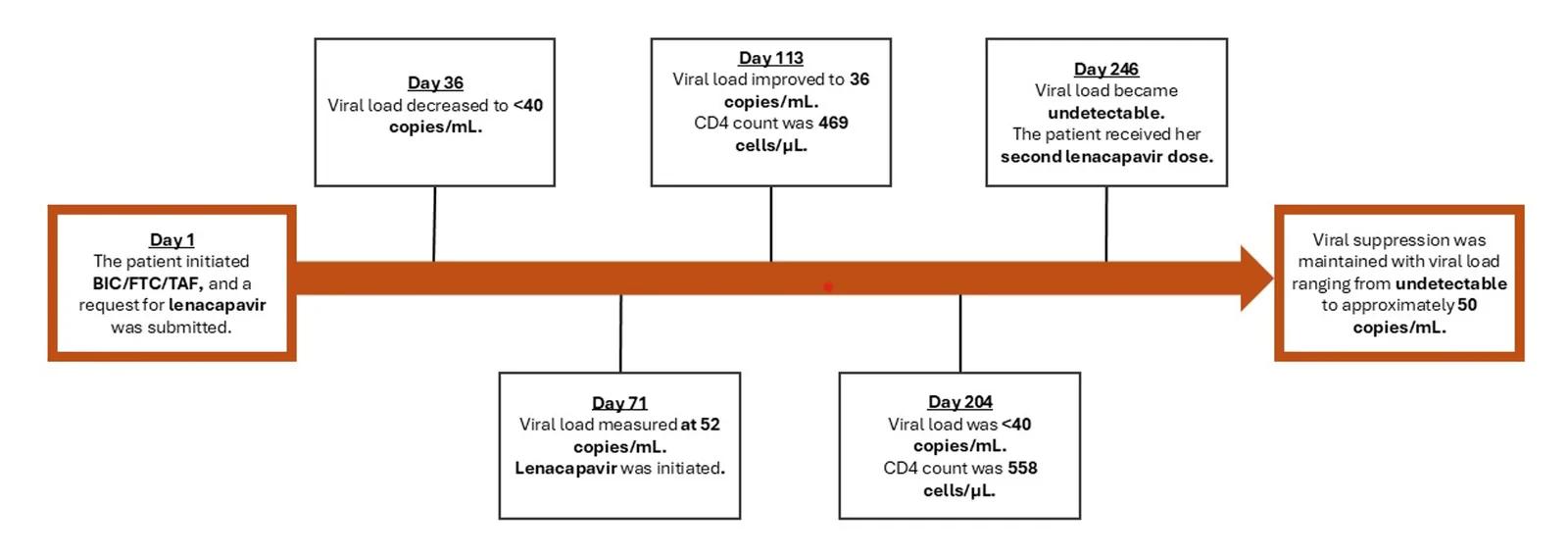
Patient virologic and immunologic monitoring BIC/FTC/TAF - bictegravir/emtricitabine/tenofovir alafenamide

## Discussion

This case report describes the patient journey associated with extensively multidrug-resistant HIV with a series of ART combinations administered in order to achieve sustained viral suppression. This eventually led to class-wide resistance to all NRTIs, all NNRTIs, and nearly all PIs, which is consistent with long-term suboptimal suppression, especially since the patient was at high risk for developing HIV drug resistance due to delayed ART initiation, the presence of resistance prior to treatment, and high baseline VL [6].

Several attempts to regain virological control using innovative or intensified strategies were undertaken over the years; however, these approaches often relied on drugs with only partial activity or were constrained by tolerability, availability, or the patient's evolving resistance profile. Consequently, the progressive accumulation of mutations significantly narrowed therapeutic options over time. In regard to recommended practice in such cases, a recent consensus document has emphasized that constructing a successful salvage regimen for HTE patients requires a highly individualized approach that accounts for comprehensive resistance profiles, previous ART history, and potential drug-drug interactions, often necessitating the inclusion of agents with novel mechanisms of action [7].

After reviewing the accumulated viral genotypes and medication history for the patient, the choice to select lenacapavir was driven by its proven efficacy in similar populations [5]. Lenacapavir, along with an optimized background regimen, was chosen based on the available guidelines [8]. The optimized background regimen in this case was selected to be BIC/FTC/TAF given no known INSTI mutations and partially active TAF in view of major transcriptase-associated mutations (TAMS), while boosted activity in the presence of M184V. Even though viral suppression was achieved after the initiation of BIC/FTC/TAF, a sustained response could not be risked with only one fully active ARV. Lenacapavir was hence added as planned, and sustained virological suppression has been maintained up to the date of this report. Similar experiences were described in other reports of real-world cases [9-13], which further support its positioning as the optimal salvage therapy in this population. However, different ART combinations were utilized in the cases available in the published literature; hence, our report adds to the body of knowledge on effective lenacapavir combinations to allow tailoring ART based on the patient's history.

This case also documents the first use of lenacapavir for HIV treatment in the UAE. Highlighting this experience would pave the way for its use in HTE patients and would minimize potential barriers to its introduction in UAE HIV clinic settings, where the lack of provider experience with newer ART regimens usually limits their use among patients eligible for them.

## Conclusions

This case demonstrates the successful salvage of multidrug-resistant HIV using lenacapavir in combination with an optimized background regimen, while securing two fully active (bictegravir and lenacapavir) and one partially active (tenofovir) antiretroviral agents, in achieving sustained virologic suppression that multiple prior regimens had failed to achieve over years of follow-up. This highlights the importance of expanding access to agents with novel mechanisms of action, such as lenacapavir, for improving outcomes in HTE populations.

For the HTE population, the integration of antiretroviral agents with novel mechanisms of action should be considered when an effective regimen cannot be constructed from conventional agents. Our case further highlights the importance of early resistance testing and continuous virological monitoring for such cases. As the population of HTE individuals with HIV continues to evolve, accumulating real-world evidence from diverse healthcare settings would contribute to more informed clinical decision-making in similar settings.
